# Deciphering Blood Flow Restriction Training to Aid Lipid Lowering in Obese College Students through Untargeted Metabolomics

**DOI:** 10.3390/metabo14080433

**Published:** 2024-08-05

**Authors:** Xianyou Cui, Sidorenko Tatiana Anatolevna, Yu Wang

**Affiliations:** 1 Zhejiang Guang Sha Vocational and Technical University of Construction, No.1 Guangfu East Street, Dongyang 322103, China; xianyou@mail.ru; 2 Moscow State Academy of Physical Education, Liubertsy District, Malakhovka, Shosseynaya St. 33, 140030 Moscow, Russia; sidtat@bk.ru; 3 Ryazan State University Named for S. A. Yesenin, St. Svobody, 46, 390000 Ryazan, Russia; 4 Moscow State University of Sport and Tourism, Kirovogradskaya Street, 21, Building 1 (South Campus), 117519 Moscow, Russia

**Keywords:** metabolic modulation, exercise prescription, lipid lowering, obese college students, blood flow restriction training

## Abstract

(1) Objective: The aim of this study was to observe the lipid-lowering effects of blood flow restriction training (BFR) combined with moderate-intensity continuous training (MICT) in obese college students by observing lipid-lowering hormones and untargeted metabolomics. (2) Methods: In this study, 14 obese college students were convened into three groups—MICT, MICT+BFR, and high-intensity interval training (HIIT)—for a crossover experiment. Blood was drawn before and after exercise for the analysis of lipolytic agents and untargeted metabolomics. The study used a paired *t*-test and ANOVA for statistical analyses. (3) Results: The lipolytic agent results showed that MICT+BFR was superior to the other two groups in terms of two agents (*p* = 0.000 and *p* = 0.003), namely, GH and IL-6 (difference between before and after testing: 10,986.51 ± 5601.84 and 2.42 ± 2.49, respectively), and HIIT was superior to the other two groups in terms of one agent (*p* = 0.000), i.e., EPI (22.81 ± 16.12). No advantage was observed for MICT. The metabolomics results showed that, compared to MICT, MICT+BFR was associated with the upregulated expression of xanthine, succinate, lactate, N-lactoylphenylalanine, citrate, ureido acid, and myristic acid after exercise, with the possibility of the involvement of the citric acid cycle, alanine, aspartic acid, glutamate metabolism, butyric acid metabolism, and the histidylate metabolism pathway. (4) Conclusions: The superior lipid-lowering effect of MICT+BFR over MICT in a group of obese college students may be due to the stronger activation of GH and IL-6 agents, with the citric acid cycle and alanine, aspartate, and glutamate metabolic pathways being associated with this type of exercise.

## 1. Introduction

According to previous studies, obesity is on the rise and has become a major public health problem that needs to be urgently addressed [1]. The hazards of obesity are persistent and varied and are associated with a series of metabolic diseases. In addition, genetic factors, overeating, poor lifestyle, lack of physical activity, and other unhealthy lifestyle choices play an important role in causing obesity [1,2,3,4]. According to a statistical survey on the prevalence of obesity, the proportion of obese individuals in one group in particular is gradually increasing, i.e., college students. This group has many significant characteristics, including the fact that students have more free time, leading to an increase in sedentary and mobile phone browsing time; engage in less physical activity; and tend to have an irregular and unhealthy diet [5].

The simple tool of BMI (body mass index) is used to differentiate between different groups of people. A BMI below 18.5 indicates that an individual is underweight, 18.5 to 24.9 is normal weight, 25 to 29.9 is overweight, and 30 and above indicates obesity. For the majority of the university student population, the transition from the role of student to that of an employee is a critical period that also marks the peak of physiological maturity and development. A lack of attention to obesity and effective interventions during this period can have a negative impact on many aspects of this group [6,7,8].

In recent years, a considerable number of stakeholders have been exploring reasonable means to address the problem of obesity among college students and increase the amount of time spent engaging in physical activity [9,10]. According to the latest research, high-intensity interval training (HIIT), as a training method that can increase body metabolism and improve body fat levels, has been hotly debated because of its short exercise time and variety of exercise forms [11]. Many studies have shown that HIIT is more effective than MICT for fat loss, partly because the higher intensity of HIIT stimulates the body to produce more lipolytic agents and partly because the recovery period after HIIT is longer, during which the body still needs to break down sugars and fats to supply energy, thus consuming more fat [11,12].

While some studies have shown that HIIT is more effective than MICT for fat loss, there are concerns that HIIT may be less palatable and not necessarily safer for obese people. This is because obese college students may feel a greater exercise burden when performing HIIT, and they may be less able to stick with such a high-intensity workout [13].

Based on this, some researchers have investigated whether it is possible to combine the blood flow restriction training (BFR) method, which is common in the field of rehabilitation, with MICT in order to obtain a combination of the high compliance and safety of MICT and the high fat-loss effect of HIIT [14]. BFR, as a widely used means in the field of rehabilitation, has been applied to patients to enhance muscle mass and muscle strength. At the same time, however, some researchers believe that BFR can also be applied to the field of aerobic exercise, i.e., using smaller aerobic loads to achieve the same workout effect as larger ones [15]. Based on this speculation, researchers believe that BFR combined with aerobic exercise has considerable advantages for fat loss in obese patients [16].

Although a handful of studies have explored the effects of BFR in combination with MICT for fat loss, few studies have made parallel comparisons between MICT, BFR+MICT, and HIIT. More importantly, few studies have delved into the possible physiological mechanisms of BFR combined with MICT for fat loss. According to previous studies, the effect of exercise on the breakdown of excess fat may be due to the increased secretion of related lipolytic agents [17,18]. Lipolytic agents, also known as lipolytics, are compounds or drugs that promote the breakdown of fat. They reduce the size and number of fat cells by increasing the breakdown of triglycerides within fat cells, converting them to free fatty acids and glycerol [19]. Lipolytics, such as hormones, caffeine, green tea extract, and alkaloids, are sometimes used in medicine to treat certain obesity conditions and are also used in the fitness and weight loss field by some groups of people as a weight loss aid [20].

Untargeted metabolomics enables the comprehensive and systematic detection of dynamic changes in all small-molecule metabolites in obese college students before and after exercise interventions [21]. This unbiased nature allows us to capture a wide range of metabolite information, thus providing a more comprehensive understanding of the effects of exercise on the metabolic systems of obese college students. Meanwhile, non-targeted metabolomics reveals the interactions and regulatory relationships between metabolites through metabolic pathway analysis, which helps us to deeply understand how exercise intervention affects the metabolic network of obese college students and provides the basis for the discovery of new metabolic pathways [22]. Finally, by analysing metabolite changes in obese college students before and after exercise interventions, non-targeted metabolomics can help us understand the differences in response to exercise interventions in different individuals. This could help to develop a more personalised exercise intervention programme to improve intervention efficacy and reduce adverse effects.

Based on this, the present study is intended to observe the effects of MICT, BFR+MICT, and HIIT on the lipid metabolism and lipid profiles of obese college students through metabolomics to explore the possible physiological mechanisms of BFR+MICT in lipid reduction in these individuals and to provide a theoretical basis for the development of exercise prescription and elaboration of the mechanism of lipid reduction in this population.

## 2. Materials and Methods

### 2.1. Study Subjects and Procedure

Fourteen obese college students were recruited throughout Jilin Normal University using posters, flyers, emails, and social networks, and the recruited subjects were required to meet the following requirements: (1) aged between 18 and 26 years; (2) BMI > 24 kg/m^2^, as defined in the Chinese Guidelines for the Prevention and Control of Overweight and Obesity in Adults [14]; (3) no regular exercise habits and no bad habits such as smoking or alcohol abuse; (4) no hormonal medications; (5) no cardiovascular, respiratory, or renal diseases; (6) no history of overweight/obesity; (7) no history of surgery in the last 6 months; and (8) no exercise-related indications. The experiment was conducted at Jilin Normal University, and all subjects were informed of the specific exercise procedures and signed an informed consent form.

In order to reduce the influence of relevant variables, the subjects were asked to do the following before the test: (1) Before and after exercise, the subjects were asked to record their food intake over a 24-h period and were asked to eat the same food (specifically, fixed window food from a selected Jilin Normal University dining hall) during that 24-h period; (2) they were told to avoid engaging in other strenuous exercise before participating in the experimental test in order to prevent any impact on the sample indexes; (3) they were asked to refrain from eating for 8 h prior to the experimental test, while the same standardised training meal was provided 1 h prior to exercise, with the content of the training meal being in line with the recommended nutrient intake; and (4) the inter-test washout period was 7 days for each group, and to control for the effects of biorhythms, the testing time was scheduled to be between 2 pm and 5 pm for all groups.

### 2.2. Study Design

**Figure 1:**
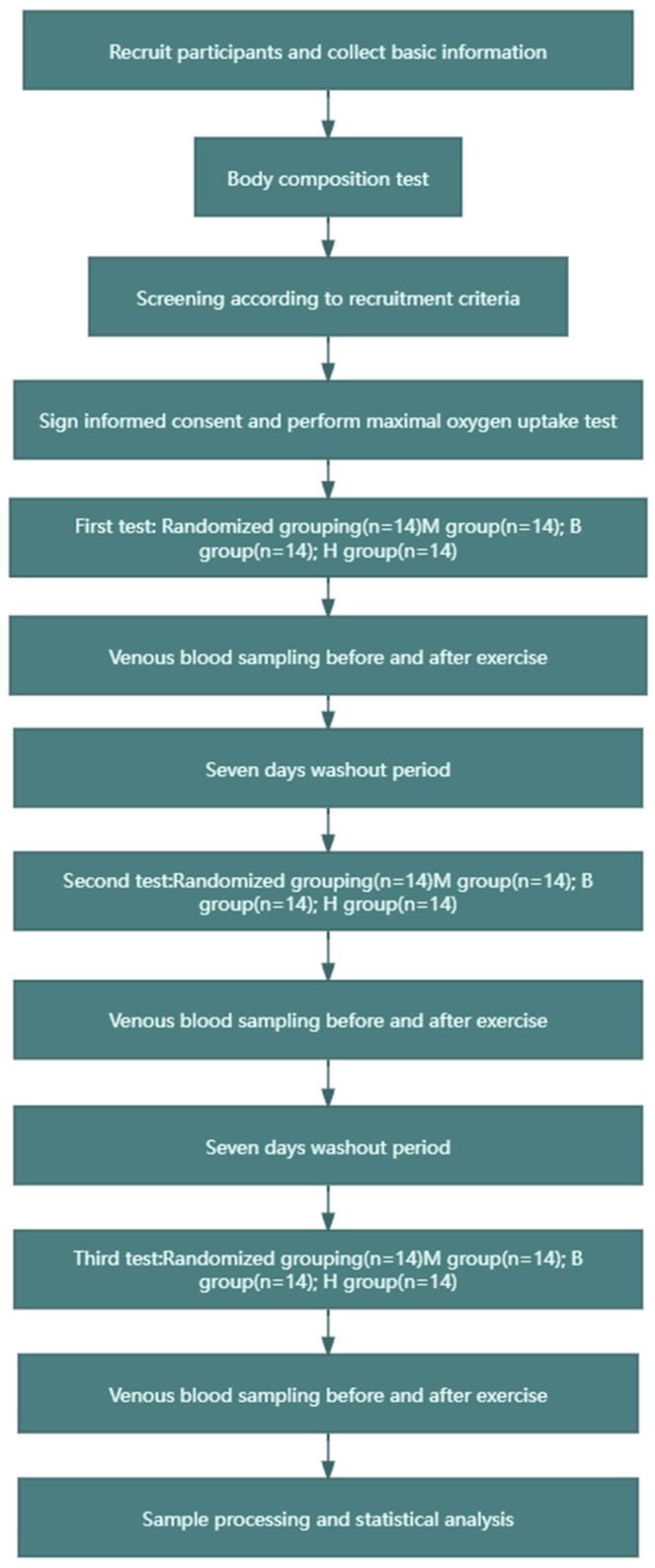
Research flow chart.

The experiment adopted a crossover design, as shown in Figure 1. The experimental group was divided into the following three subgroups, to which the recruited subjects were randomly assigned: (1) the MICT group (60% VO2max intensity, 200 KJ of exercise, no BFR), (2) the MICT+BFR group (60% VO2max intensity, 200 KJ of exercise, 60% limb occlusion pressure BFR), and (3) the HIIT group (85% VO2max intensity, 200 KJ of exercise, 60% BFR of limb occlusion pressure). In this case, both 60% VO2max and 85% VO2max intensity values were determined based on a linear regression between VO2 and power output in maximal oxygen uptake tested prior to the experiment, and after determination, the total time taken to complete 200 KJ for both exercise intensities was calculated (200 KJ of work carried out/exercise carried out). In addition, the MICT group and the MICT+BFR group both exercised continuously, while the HIIT group exercised for 3 min with a passive interval of 3 min until 200 KJ of mechanical work was completed. The subjects in the MICT group, the MICT+BFR group, and the HIIT group underwent a pre-elbow venous blood sampling of 5 mL immediately before and immediately after exercise, and the samples were analysed, extracted, and compared for each of the correlation indexes [23].

In the protocol design, the MICT group was the control group of this experiment, the MICT+BFR group was the main experimental group studied in this experiment, and the HIIT group was the exercise control group. In the detection and analysis of non-targeted metabolomics, in order to study the core problem of blood flow limitation, only the indicators in the blood of the subjects in the MICT group and the MICT+BFR group were compared and analysed using non-targeted metabolomics.

### 2.3. Body Composition Test

First, the subjects were required to stand barefoot in an upright position on a test bench for the height test. During the test, the shoulders, back, and hips were pressed against the height measuring tape (minimum scale of 1 mm), and the measurement values were read and recorded by the measurement personnel after the measurement was completed. Subsequently, a bioelectrical impedance analyser (Yunkangbao, Shenzhen, China) was used to test the subjects’ body composition, with the subjects wearing light clothing and standing barefoot on metal electrodes. The main indicators of the test were the body mass index (BMI) and the percentage of body composition (Fat%). The subjects were asked to take the measurements on an empty stomach in the morning after a night’s rest, the Chinese Inbody Body Composition Test Instrument was used.

### 2.4. Maximum Oxygen Uptake Test

An incremental load gradient test was used, with the load intensity increasing every 2 min, and VO2 was recorded and assessed using a gas metabolism analyser throughout the test, with mean values extracted every 30 s. First, the subject warmed up using an aerobic power bike, and a respiratory mask was fitted and worn on the subject’s face after steady breathing. Secondly, a heartrate belt was attached to the subject’s skin and worn at the same height as the heart while ensuring that the heartrate belt’s sensing area was located near the heart. Finally, the gas metabolism analyser was switched on and connected to the respiratory mask to ensure that it was airtight throughout the test [24]. For male subjects, an aerobic power train of 100 W was used as the initial loading intensity, with 30 W increments every 2 min; for female subjects, an aerobic power train of 50 W was used as the initial loading intensity, with 25 W increments every 2 min. The RPM of the aerobic power train was always kept within the range of 60 ± 5 r/min throughout the whole testing process, and the test was stopped when the subjects were exhausted. During recording and analysis, VO2max was the highest mean value within 30 s. The instruments used in this part of the study were a aerobic power bike (Monark LC6, Swedish) a Finnish heartrate belt (H10, Polar) and a gas metabolism analyser (Cosmed Quark-PFT4. Italian).

The criteria [25] for assessing the subject’s exhaustion in the maximal oxygen uptake test were as follows: (1) the aerobic power vehicle (Jiafu, Shanghai, China) RPM could not be maintained within the range of 60 ± 5 r/min, and the heart rate reached more than 180 BPM; (2) the subject could no longer continue the exercise after encouragement; (3) the intensity of the exercise was no longer linearly related to the oxygen uptake when the exercise load was continuously increased; (4) the respiratory quotient was more than 1.15; and (5) there was evidence of the development of exercise stress syndrome.

### 2.5. Sample Collection and Processing

Blood was collected from the anterior elbow vein in all groups before and immediately after each exercise session [26]. The procedure was conducted as follows: 5 mL of venous blood was drawn from the anterior elbow vein via venipuncture from a heparin anticoagulant tube while the participant was sitting on the collection table at rest before exercise and immediately after exercise. Subsequently, the blood collection tubes were weighed and compared to ensure that the centrifuge speed was balanced. After comparison, the anticoagulated tubes were placed in the centrifuge and centrifuged at 2000× *g* for 10 min at a temperature of 4 °C. At the end of the centrifugation, the upper plasma layer was extracted with a pipette gun and stored in a −80 °C freezer.

### 2.6. Lipolytic Agent Test

A competitive enzyme immunoassay [27] was used to detect epinephrine (EPI) and noradrenaline (NA) levels. EPI and NA ELISA kits (CUSABIO, Wuhan Huamei Bio, Wuhan, China) were used.

A double-antibody sandwich enzyme-linked immunosorbent assay (ELISA, Mlbio, Shanghai, China) [28] was used to detect growth hormone (GH) and IL-6 content. The assay was performed using a GH and IL-6 ELISA kit (MultiSciences, Hangzhou, China).

### 2.7. Non-Targeted Metabolomics Testing

#### 2.7.1. Metabolic Sample Processing

The preprocessing of metabolic samples consisted of the following four steps. First, the separated samples were lyophilised using a lyophiliser. Subsequently, the lyophilised samples were ground to a powdered form using a cryo-mixer grinder (Retsh MM400, Heidelberg, Germany) containing zirconia beads for 1 min at 35 Hz. Next, approximately 100 mg of the ground powdered sample was taken and mixed with a 70% concentration of methanol to obtain 0.1 g/mL aqueous solution, and the mixed aqueous sample was sonicated at 40 Hz. Finally, metabolites were extracted by centrifugation and filtration [29].

#### 2.7.2. Detection and Analysis of Metabolic Samples

The non-targeted metabolomics analysis of all plasma samples before and after exercise in the MICT group and the MICT+BFR group was performed using LC-MS/MS (EXPEC, Shanghai, China). The analysis was performed using an electrospray ion source with the following ion source parameters: nebulising gas flow rate of 3 L/min, heating gas flow rate of 10 L/min, DL temperature of 250 °C, ambient temperature of 500 °C, heat-block temperature of 400 °C, and drying gas flow rate of 10 L/min [30].

The analyses were performed using Compound Discoverer 3.3 software to compare the MS2 detection spectra with high-quality metabolic signals with S/N > 10 with the database information, and the compared metabolic signals were batch-annotated. Subsequently, metabolic signals that did not match those in the database were identified and searched for in the literature and online databases. Finally, retention times (RTs), precise *m*/*z* values, and fragmentation patterns obtained by injecting standards under similar conditions were compared to identify standard-containing compounds [31].

#### 2.7.3. Visualisation Method

In order to explain the results more clearly, this study used pictures to visualise the results, and the main research methods and implications are reported as follows [32].

The PLS-DA overview plot and PLS-DA multivariate statistical score plot: This is a model obtained using MetaboAnalyst normalisation, using the fold-change (FC) value as a construct variable for metabolite analysis and screening. PLS-DA, as a supervised and efficient analytical method, is capable of modelling the relationship with the sample subgroups based on subgrouping information at the time of analysis to obtain better separation effects between groups.

Hotspot map of plasma metabolite: A heat map represents a matrix of data, where differences between the data are visualised through the use of a colour gradient and where larger differences are retained while smaller differences are highlighted through data scaling. Different coloured regions represent different cluster-grouping information, where metabolic patterns within the same group are similar and may have similar functions or participate in the same biological process. Therefore, by clustering metabolites with the same or similar metabolic patterns into groups, the biological functions of known or unknown metabolites can be inferred.

Volcano plot of pre-exercise plasma metabolite: The results of the analysis of the FC values were plotted as horizontal coordinates, and the results of the sample *t*-tests were plotted as vertical coordinates so as to screen the important metabolites by biological significance and statistical significance. Significantly expressed metabolites were screened by using an FC ≥ 1.5 or ≤0.67 with a *p*-value < 0.05, with those closer to the top representing more significant differences.

Score graph of VIP scores: Variable Importance in Projection scores (VIP scores) are metrics used in metabolomics to assess the categorical importance of different metabolites to a sample, and they are usually used to screen for marker metabolites with a VIP value >1.

Bubble diagram of metabolic pathways: The vertical coordinate is the name of the metabolic pathway, and the horizontal coordinate is the *p*-value. The significance of the *p*-value is to determine whether there is sample enrichment in a certain pathway; the smaller the *p*-value, the darker the colour, which means that the sample enrichment in the pathway is more significant, as usually signified by *p* < 0.05. In the enrichment ratio, bigger bubbles mean that the number of enriched metabolites is greater.

### 2.8. Data Analysis

The lipolytic agent data reported in this study were statistically analysed using SPSS 26.0, with the data described as mean ± standard deviation (Mean ± SD), and the experimental data were tested for normality using the Shapiro–Wilk normality test. A paired-samples *t*-test was used to compare the pre- and post-exercise data within each group for the statistical analysis of the changes in lipolytic agent data before and after exercise, and repeated-measures analysis of variance (ANOVA) was used to statistically analyse the changes in the lipolytic agent data between the groups, with *p* < 0.05 representing a statistically significant difference and *p* < 0.01 representing a more significant statistical difference. In addition, changes and differences in lipolytic agents within and between groups were plotted and described using GraphPad Prism 9.5.

The metabolomics data from this study were uploaded to the MetaboAnalyst 6.0 web platform (https://www.metaboanalyst.ca/ (accessed on 30 May 2024)) as a complete dataset containing chemical names or human metabolome database IDs in csv format. In preprocessing the data, missing values were replaced using NKK (feature-wise), data were filtered using interquartile range (IQR), and sample data were normalised using logarithmic transformation (base of 10) and Pareto scaling (centred on the mean and divided by the square root of the standard deviation of each variable). Multivariate statistical analyses were performed using partial least squares discriminant analysis (PLS-DA) to determine sample variability before, during, and after exercise and between groups. Variable importance projection scores (VIP scores) were used to screen for marker metabolites that were important for model separation using a VIP value >1. Differential metabolite changes were represented by hotspot and volcano plots, and screening criteria were based on a fold change (FC) ≥ 1.5 or ≤0.67, with a *p*-value < 0.05 used for screening.

## 3. Results

### 3.1. Comparison of the Effects of Three Forms of Exercise on the Levels of Different Lipolytic Agents

#### 3.1.1. Comparison of EPI Levels before and after Exercise in MICT, HIIT, and MICT+BFR Groups

It was found that (1) there was no statistically significant difference in EPI levels among the groups before exercise (*p* > 0.05); (2) compared with the pre-exercise EPI levels, the difference in blood EPI levels among the subjects in the MICT and MICT+BFR groups after exercise was significant (*p* < 0.05), with increases of 8.3% and 18.1%, respectively; and (3) compared with the pre-exercise EPI levels, for the HIIT group, the difference in blood EPI levels in subjects after exercise was highly significant (*p* < 0.01), with an increase of 30.4%, as shown in Table 1. When intergroup comparisons were made, it was found that (1) the intergroup difference in blood EPI levels between the subjects in the HIIT group and those in the MICT group was highly significant (*p* < 0.01); (2) the intergroup difference in blood EPI levels between the subjects in the MICT+BFR group and those in the MICT group was not significant (*p* > 0.05); and (3) the difference in blood EPI levels between the subjects in the HIIT group and those in the MICT+BFR group was highly significant (*p* > 0.05). The intergroup differences in blood EPI levels were not significant (*p* > 0.05), as shown in Table S1.

#### 3.1.2. Comparison of NA Levels before and after Exercise in MICT, HIIT, and MICT+BFR Groups

It was found that there was no statistically significant difference in the NA levels of the groups before exercise (*p* > 0.05). Compared with the NA levels before exercise, the differences in the NA levels in the blood of the subjects in the MICT group, the HIIT group, and the MICT+BFR group after exercise were not significant (*p* > 0.05), with increases of 4.8%, 7.4%, and 9.7%, respectively, as shown in Table 2. When intergroup comparisons were made, it was found that none of the intergroup differences in subjects’ blood NA levels between the MICT, HIIT, and MICT+BFR groups were significant (*p* > 0.05), as shown in Table S2.

#### 3.1.3. Comparison of GH Levels before and after Exercise in MICT, HIIT, and MICT+BFR Groups

No statistically significant difference was found in the GH levels among the groups before exercise (*p* > 0.05). Compared with the pre-exercise GH levels, the differences in blood GH levels among the subjects in the MICT, HIIT, and BFRT groups after exercise were highly significant (*p* < 0.01), with increases of 3309%, 3988%, and 5014%, respectively, as shown in Table 3. When intergroup comparisons were made, it was found that (1) compared with the MICT group, the intergroup differences in the blood GH levels among subjects in the HIIT group were not significant (*p* > 0.05); (2) compared with the MICT group, the intergroup differences in the blood GH levels among subjects in the MICT+BFR group were highly significant (*p* < 0.01); (3) the differences in blood GH levels among subjects in the HIIT group and the MICT+BFR group were highly significant (*p* < 0.01); and (4) the intergroup differences in blood GH levels between subjects in the HIIT group and the MICT+BFR group were not significant (*p* > 0.05), as shown in Table S3.

#### 3.1.4. Comparison of IL-6 Levels before and after Exercise in MICT, HIIT, and MICT+BFR Groups

It was found that (1) there was no statistically significant difference in IL-6 levels among the groups before exercise (*p* > 0.05); (2) compared with the pre-exercise IL-6 levels, the difference in IL-6 levels in the blood of the subjects in the MICT group after exercise was not significant (*p* > 0.05), with an increase of 14.1%; (3) compared with the pre-exercise IL-6 levels, the difference in the blood of the subjects in the HIIT group after exercise was significant (*p* > 0.01); (4) compared with the pre-exercise IL-6 levels in the HIIT group, after exercise, the difference in the blood was significant (*p* > 0.05), with an increase of 35.1%; and (5) compared to pre-exercise IL-6 levels, the difference in post-exercise blood IL-6 levels of subjects in the MICT+BFR group was highly significant (*p* < 0.01), with an increase of 52.4%, as shown in Table 4. When intergroup comparisons were made, it was found that none of the intergroup differences in subjects’ blood IL-6 levels between the MICT, HIIT, and MICT+BFR groups were significant (*p* > 0.05), as shown in Table S4.

**Figure 2:**
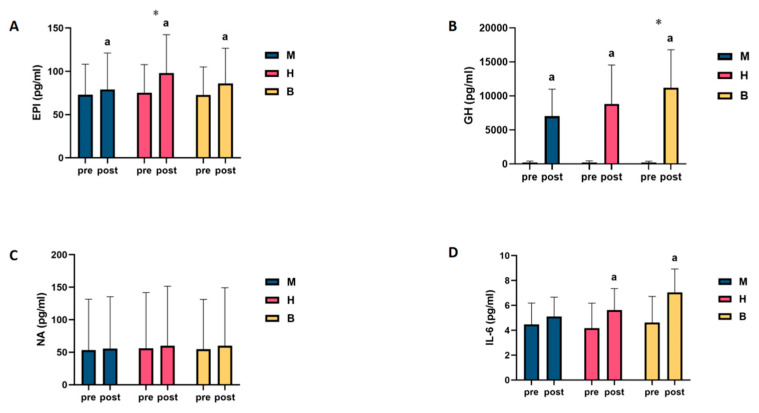
Intra- and intergroup comparisons of levels of lipolytic agents before and after exercise in MICT, HIIT, and MICT+BFR groups. (**A**): EPI; (**B**): GH; (**C**): NA; (**D**): IL-6; a: within-group comparison is significant; *: between-group comparison is significant. M: MICT, H: HIIT, B: MICT+BFR.

Pooling and analysing the above results revealed that only in NA was a lipolytic agent found, and no change in the level of this agent was found as a result of exercise, regardless of the type of exercise. However, in the IL-6 test, it was found that only MICT was unable to drive changes in the levels of this agent. In the GH and IL-6 tests, it was found that MICT+BFR may be superior to the other two exercise regimens, while only the EPI test showed that HIIT was superior to the other two exercise regimens, as shown in Figure 2.

### 3.2. Metabolomic Analysis of MICT and MICT+BFR

#### 3.2.1. Comparison of Metabolic Characteristics before and after Exercise in MICT Group

The sample aggregation before and after exercise in the MICT group was better, with a first principal component (principal component 1, PC1) of 24.6% and a second principal component (principal component 2, PC2) of 14%, showing a high degree of separation between QM (before exercise in the MICT group) and HM (after exercise in the MICT group).

The 15 metabolites with VIP > 1 before and after exercise in the MICT group were propionylcarnitine, L-argininosuccinic acid, L-valine, lactic acid, succinic acid, malic acid, fumaric acid, acetoacetic acid, p-xanthine, pyruvate, xanthouric acid, n-leucine, acetyl L-carnitine, uric acid, and valine, which were considered to be the most important metabolites in the separation of the model of the MICT group. The four compounds with the highest scores were considered to be metabolites that were extremely important for the isolation of metabolic changes before and after exercise in the MICT group (propionylcarnitine, L-argininosuccinic acid, L-valine, and lactic acid).

A differential hotspot analysis of plasma metabolites before and after exercise in the MICT group revealed that a total of 15 different metabolites were extracted (TOP15), of which 12 metabolites were upregulated and 3 were downregulated. Among the extracted metabolites, a total of 10 significantly expressed metabolites were screened (malic acid, N-lactoylphenylalanine (Lac-Phe), succinic acid, L-argininosuccinic acid, fumaric acid, acetoacetic acid, propionylcarnitine, xanthouric acid, p-xanthine, and lactate), and all of these 10 significantly expressed metabolites were upregulated post-exercise when compared to pre-exercise.

Finally, enrichment analysis was performed, which showed that the metabolic pathways with *p* < 0.05 mainly included the citric acid cycle (TCA cycle), pyruvate metabolism, arginine biosynthesis, butyric acid metabolism, alanine, aspartate and glutamate metabolism, and ketone body synthesis and degradation.

#### 3.2.2. Comparison of Metabolic Characteristics before and after Exercise in MICT+BFR Group

The sample aggregation was better in the MICT+BFR group before and after exercise, with 39.1% for PC1 and 17.5% for PC2, showing a high degree of separation between QB (pre-exercise in the MICT+BFR group) and HB (post-exercise in the MICT+BFR group).

The 15 metabolites (lactate, L-valine, succinate, fumarate, acetyl L-carnitine, propionyl carnitine, choline, pyruvate, malate, n-leucine, levulinic acid, acetoacetate, L-arginine aminosuccinic acid, xanthurenic acid, and lecithin) with VIP values > 1 for variable importance before and after exercise in the MICT+BFR group were considered the most important metabolites in the separation of the model in the MICT+BFR group. The top four compounds with the highest VIP scores were considered extremely important for the separation of pre- and post-exercise metabolic changes in the MICT+BFR group (lactic acid, L-valine, succinic acid, and fumaric acid).

A differential hotspot analysis of pre- and post-exercise plasma metabolites in the MICT+BFR group revealed that a total of 15 differential metabolites (TOP15) were extracted, of which 13 were upregulated and 2 were downregulated.

Finally, enrichment analysis was performed, and the results showed that the metabolic pathways with *p* < 0.05 mainly included the citric acid cycle (TCA cycle), pyruvate metabolism, glycolysis/glycogen isomerisation, alanine, aspartate and glutamate metabolism, and the synthesis and degradation of ketone bodies.

#### 3.2.3. Comparison of Pre-Exercise Metabolic Characteristics between MICT Group and MICT+BFR Group before Exercise

**Figure 3:**
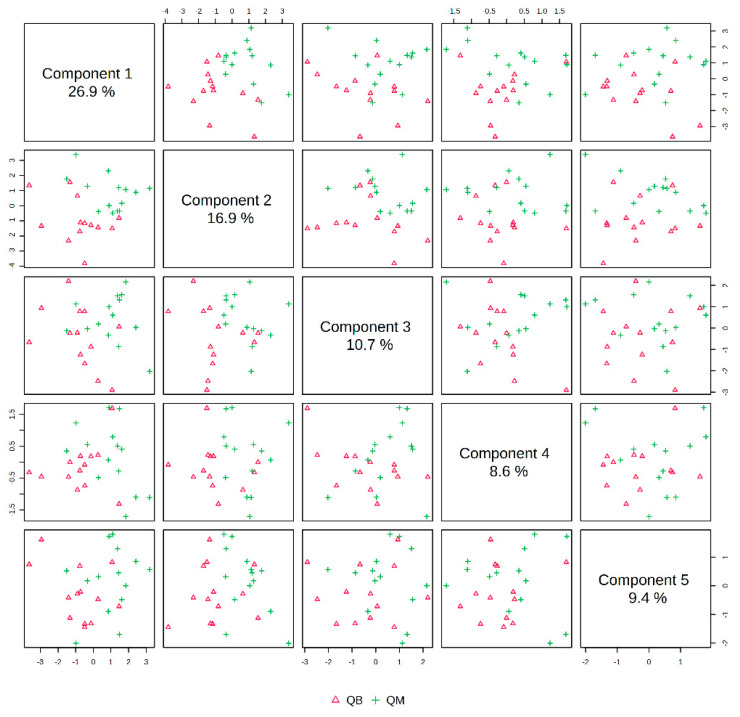
Overview of PLS-DA before exercise for both groups. Note: QM indicates pre-exercise for the MICT group; QB indicates pre-exercise for the MICT+BFR group.

**Figure 4:**
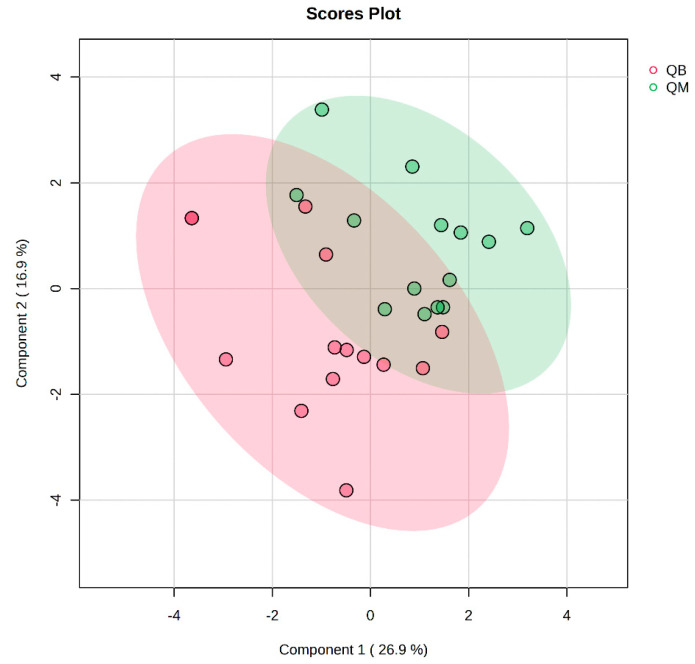
Plot of PLS-DA multivariate statistical scores before exercise for both groups. QM (pre-exercise in MICT group), QB (pre-exercise in MICT+BFR group).

The pre-exercise PLS-DA overview plots and PLS-DA multivariate statistical score plots of the MICT group and MICT+BFR group after MetaboAnalyst normalisation are shown in Figure 3 and Figure 4. The two models show different metabolic behaviours of the pre-exercise samples of the two groups, and the models use the fold change (FC) value as a construct variable for metabolite analysis and screening.

Figure 2 presents the PLS-DA score plot, showing better aggregation of pre-exercise samples in the two groups—26.9% in PC1 and 16.9% in PC2—but does not show a significant separation between QM (pre-exercise in MICT group) and QB (pre-exercise in MICT+BFR group).

**Figure 5:**
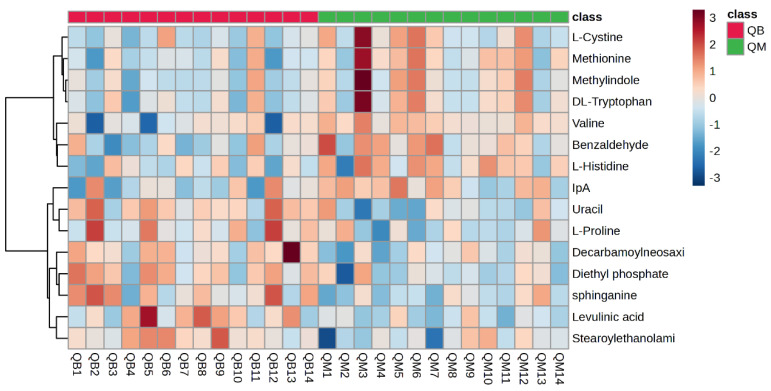
Hotspot map of plasma metabolite differences between the two groups before exercise. Note: QM indicates pre-exercise in the MICT group; QB indicates pre-exercise in the MICT+BFR group.

Figure 5 shows a hotspot map of the pre-exercise plasma metabolite differences between the MICT group and the MICT+BFR group using Ward’s clustering method and the Euclidean measurement method. The red positive value on the right side of the figure represents upregulation, and the blue negative value represents downregulation. A volcano map of the pre-exercise plasma metabolite differences between the MICT group and the MICT+BFR group is shown in Figure 5. Combining Figure 3 and Figure 4 shows that the two groups were not screened for significantly different metabolites before exercise.

#### 3.2.4. Post-Exercise Metabolic Characteristics of the MICT Group Compared to the MICT+BFR Group after Exercise

**Figure 6:**
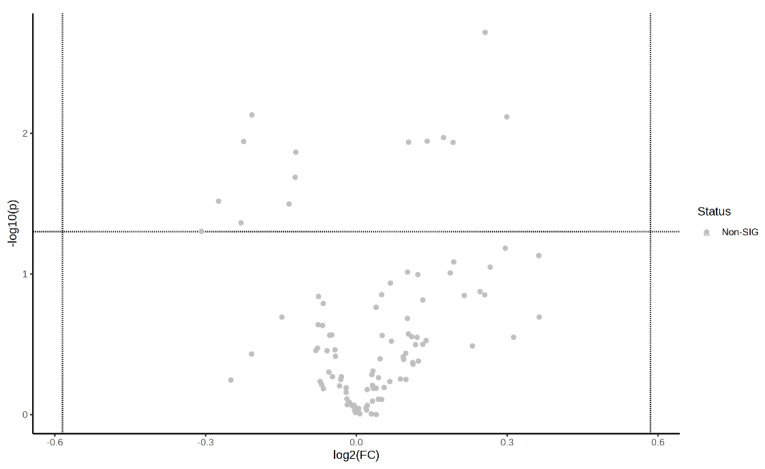
Volcano plot of pre-exercise plasma metabolite differences between the two groups.

The post-exercise PLS-DA overview plots and PLS-DA multivariate statistical score plots for the MICT group and the MICT+BFR group after MetaboAnalyst normalisation are shown in Figure 5 and Figure 6. The two models show different metabolic behaviours of the samples from the two groups post exercise, and the models use the FC value as a constructed variable for the metabolite analysis and screening.

**Figure 7:**
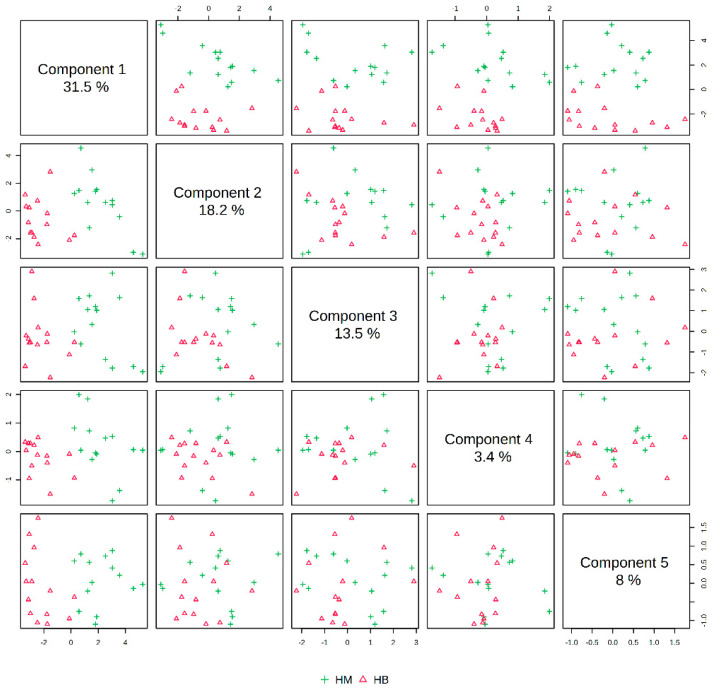
Overview of PLS-DA after exercise in both groups. Note: HM indicates the MICT group post exercise; HB indicates the MICT+BFR group post exercise.

In Figure 7, the PLS-DA score plot shows better aggregation of the samples after exercise in both groups, with 31.5% for PC1 and 18.2% for PC2, showing a more pronounced separation between HM (after exercise in the MICT group) and HB (after exercise in the MICT+BFR group).

**Figure 8:**
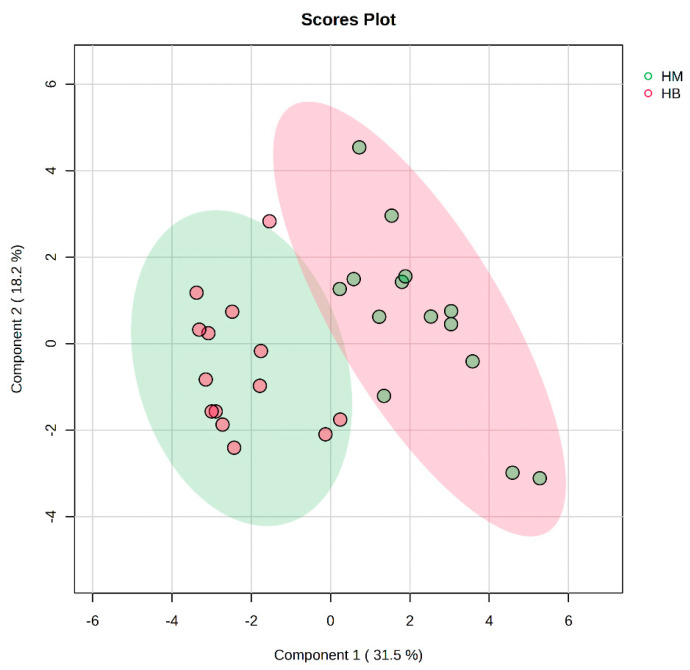
PLS-DA multivariate statistical score plot after exercise for both groups. HM indicates the MICT group post exercise; HB indicates the MICT+BFR group post exercise.

A plot of the VIP scores after exercise in the MICT group and after exercise in the MICT+BFR group is shown in Figure 8, which shows the 15 metabolites (lactate, choline, succinate, L-carnitine, pyruvate, fumarate, xanthine, lecithin, acetyl L-carnitine, L-valine, myristic acid, DL-tryptophan, L-cystine, and L-cystine) that had a post-exercise variable significance > 1 in both groups. The top four compounds with the highest VIP scores were considered to be metabolites that were extremely important for the separation of post-exercise metabolic changes between the two groups (lactate, choline, succinate, levulinic acid, and pyruvate).

**Figure 9:**
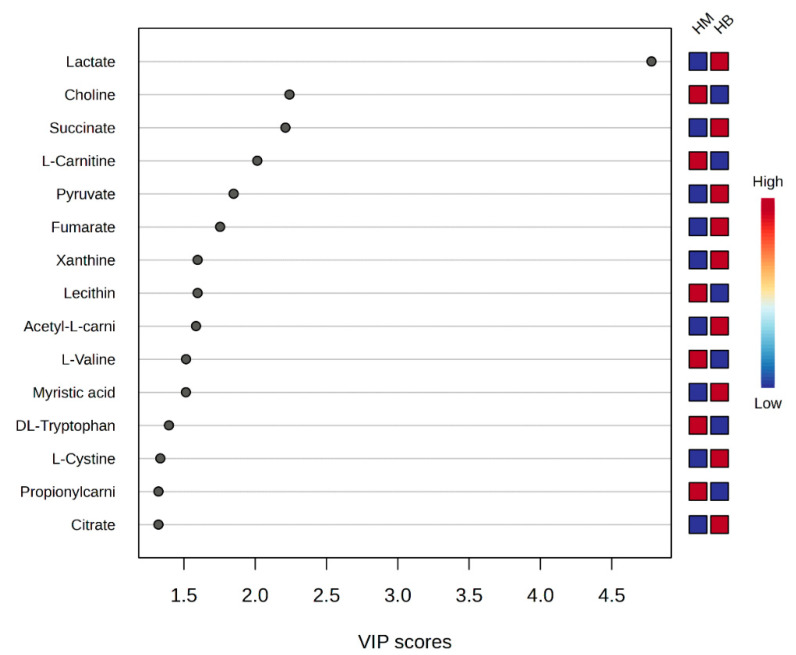
Score graph of VIP scores after exercise in both groups. Note: HM indicates post exercise in the MICT group; HB indicates post exercise in the MICT+BFR group; the right side indicates the metabolic levels (high–low) after exercise in both groups.

The plasma metabolite difference hotspot map after exercise in the MICT group and the MICT+BFR group is shown in Figure 9 using Ward’s clustering method and the Euclidean measurement method. Compared with the post exercise of group M, the red positive value on the right side of the picture represents upregulation, and the blue negative value represents downregulation. The results showed that a total of 15 different metabolites were extracted (TOP15), among which 12 metabolites were upregulated and 3 were downregulated.

**Figure 10:**
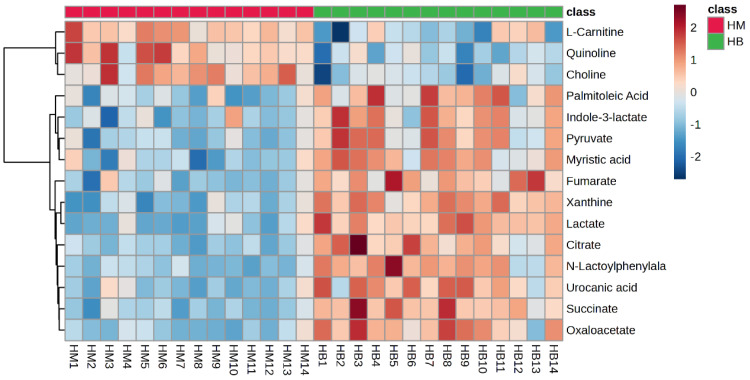
Hotspot map of plasma metabolite differences between the two groups after exercise. Note: HM indicates post exercise in the MICT group; HB indicates post exercise in the MICT+BFR group.

**Figure 11:**
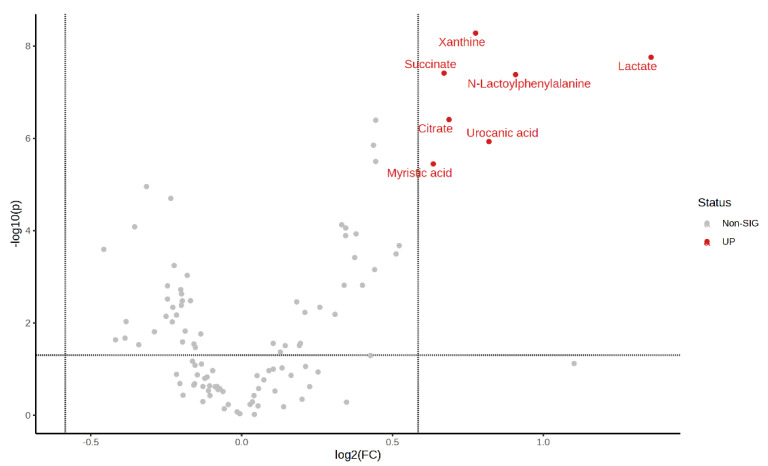
Volcano plot of plasma metabolite differences between the two groups after exercise.

Volcano plots of the plasma metabolite differences between the post-exercise plasma metabolites in the MICT group and the MICT+BFR group are shown in Figure 10 and Figure 11, which were plotted using the results of the analysis of the FC value as the horizontal coordinate and the results of the *t*-test as the vertical coordinate. The significantly expressed metabolites were screened according to FC ≥ 1.5 or ≤0.67, with a *p*-value of <0.05. The results showed that among the extracted metabolites, a total of seven significantly expressed metabolites were screened (xanthine, succinic acid, lactic acid, N-lactoylphenylalanine (Lac-Phe), citric acid, ureidoacetic acid, and myristic acid), and all were upregulated after exercise in the MICT+BFR group compared with the MICT group.

**Figure 12:**
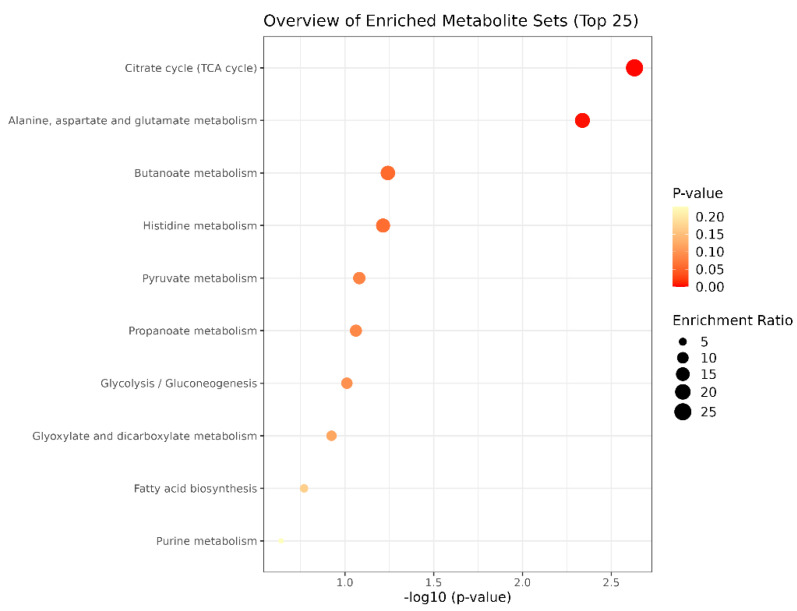
Bubble diagram of metabolic pathways after exercise in both groups.

Bubble plots of metabolic pathways after exercise in the MICT group and the MICT+BFR group are shown in Figure 12, with the names of the metabolic pathways on the vertical coordinates and the *p*-values on the horizontal coordinates. For the enrichment ratio, larger bubbles represent a higher number of enriched metabolites. The results showed that the metabolic pathways with *p* < 0.05 mainly included the citric acid cycle (TCA cycle), alanine, aspartate and glutamate metabolism, butyric acid metabolism, and histidylate metabolism.

## 4. Discussion

EPI and NA are secreted by the adrenal medulla and the sympathetic nervous system (SNS). EPI and NA stimulate lipolysis and ameliorate metabolic disorders by binding to adrenergic receptors (α and β) [33]. EPI, as an important agent for the regulation of physiological activity in the body, is functionally regulated in different states of the organism (resting and exercising), and it has been shown that EPI increases significantly during exercise [34]. However, the magnitude of the increase in concentration is influenced by a variety of factors, such as the type of exercise, the intensity and duration of exercise, and the individual’s BMI level [35,36]. Consistent with previous studies, MICT in combination with BFR achieved a pro-secretory effect that could not be achieved with MICT alone but was not as effective as HIIT [37]. Based on this, we suggest that EPI may require a sufficiently high intensity of exercise to achieve better secretion in the obese population, but at the very least, the simple training method of increasing BFR alone can achieve both safety and efficacy gains that cannot be achieved with MICT alone.

More importantly, the present study also found that MICT+BFR seems to be more effective for two lipolytic agents, namely GH and IL-6, the latter of which is a peptide agent containing 191 amino acids secreted by the anterior pituitary gland and is an important regulator of the metabolism of a variety of substances in the human body. It not only promotes the growth and development of the body and the growth and reproduction of tissues, organs, and systems but also activates protein synthesis [38]. Likewise, GH can not only promote the growth and development of the body and the enlargement and reproduction of various tissues, organs, and systems, as well as protein synthesis, but can also activate agent-sensitive lipase, increase the utilisation of fat, and promote fat decomposition [36,39,40]. There is currently a large number of research results showing that exercise can promote the body’s secretion of GH, but the amount of GH released also depends on the intensity and type of the exercise involved; compared with low-intensity exercise, high-intensity exercise can cause a significant increase in the concentration of GH [40,41]. In this study, there was no significant difference in GH levels between the groups before exercise, and the GH concentrations increased significantly in all groups after exercise compared. The increase in GH in the MICT+BFR group was significantly higher than that in the MICT group and the HIIT group. IL-6 is a single polypeptide chain of multidirectional cytokines consisting of 185 amino acids that can regulate inflammation, immunity, and host defence, in addition to acting as an energy distributor in the muscle tissue to maintain the body’s energy [42]. The relevance of IL-6 to obesity is demonstrated by the fact that IL-6 secreted during exercise can provide energy by activating AMP-activated kinases and enhancing glucose catabolism while promoting fat oxidation and lipolysis [43]. After exercise, in contrast to the MICT group, the IL-6 concentrations in the HIIT and MICT+BFR groups increased significantly compared with the pre-exercise period, and although the difference between the groups did not reach statistical significance after exercise, the magnitude of the IL-6 increase in the MICT+BFR group was higher than that in the MICT and HIIT groups. This is in line with the results of a similar study by Zheng et al. [40], who concluded that lower-intensity aerobic exercise in combination with BFR resulted in a more significant improvement in glycolipid metabolism and inflammatory markers (FPG, HbA1c, HOMA-IR, and FFA) in patients with T2DM. Based on the above, our study suggests that when prescribing exercise for obese college students, BFR is a training modality that can be considered and applied in order to take into account both safety and effectiveness.

In order to further explore the physiological mechanism of MICT+BFR to improve lipid metabolism in obese college students, the present study included a metabolomic analysis of the MICT and MICT+BFR groups under the same workload using liquid chromatography–mass spectrometry (LC-MS/MS). This analysis was conducted in order to investigate the effects of BFR on metabolites and metabolic pathways in overweight/obese college students and to provide new ideas for exercise to improve the current situation of overweight/obesity in this population.

The results of this study showed that there were no obvious metabolites in the MICT group or the MICT+BFR group before exercise. The main metabolites in the MICT and MICT+BFR groups after exercise were xanthine, succinic acid, lactic acid, N-lactoyl phenylalanine (Lac-Phe), citric acid, uremic acid, and myristic acid [44]. The main metabolic pathways were the citric acid cycle (TCA cycle), alanine, aspartate and glutamate metabolism, butyric acid metabolism, and histidylate metabolism [45]. Purines have important roles in neural regulation and transmission, cell proliferation and differentiation, and energy metabolism. Xanthine is a purine base that is widely distributed in organs and fluids of the human body, as well as other organisms, and is commonly used as a mild stimulant or in the treatment of asthma. The synthesis and conversion of xanthine is related to purine metabolism. In purine metabolism, adenine ribonucleotide (AMP), guanine ribonucleotide (GMP), hypoxanthine ribonucleotide (IMP), and xanthine ribonucleotide (XMP) all shed their amino groups to form xanthine, which ultimately produces uric acid as a potent antioxidant in the organism under the action of xanthine oxidase (XO) [46,47,48,49]. It has been suggested that plasma xanthine oxidoreductase activity is associated with visceral fat accumulation and physical inactivity and that aerobic exercise training decreases plasma xanthine oxidoreductase activity. Meihua et al. performed a metabolomic analysis of 28 sweat samples from 14 long-distance runners before and after exercise fatigue using LC-MS, which showed an upregulation of differential metabolites such as xanthine and pyruvate, suggesting that the main metabolic pathways involved in the production of differential metabolites were purine and amino acid metabolism.

Citric acid, together with succinic acid, malic acid, and fumaric acid, is an intermediate of the TCA cycle, which is the final common oxidative pathway for carbohydrates, fats, and amino acids and is the most important metabolic pathway for the body’s energy supply. It also plays an important role in gluconeogenesis, transamination, and deamination, as well as in adipogenesis [50]. Throughout the stages of the TCA cycle, first, acetyl coenzyme A and oxaloacetate are catalysed by citrate synthase to synthesise citric acid, followed by the conversion of citric acid as a substrate to aconitic acid, which is dehydrated and rehydrated to produce isocitric acid. Isocitric acid, in turn, produces oxaloacetate in a series of catalytic and transformational processes; then, finally, a new round of the TCA cycle is initiated [51,52]. The whole cycle is concluded with the regeneration of oxaloacetic acid, with catalytic transformations of succinic acid, fumaric acid, and malic acid in between. The whole chemical process is capable of generating two-thirds of the food energy source; thus, in combination with the above analyses, as well as the results of the present study, it is clear that the TCA cycle is an extremely important metabolic pathway in organisms [53,54].

The main differential metabolites of MICT and MICT+BFR after exercise in this study were N-lactoylphenylalanine (Lac-Phe), urocanic acid, and myristic acid, of which Lac-Phe has been mentioned in previous analyses as a novel “exercise factor” related to appetite, a pseudo-dipeptide generated from lactate and phenylalanine, and that can stimulate the production of Lac-Phe in the organism [55]. The production of Lac-Phe can be stimulated by exercise, e.g., the release of lactate from skeletal muscle during high-intensity exercise leads to a surge in circulating lactate-derived pseudo-dipeptide metabolites, including Lac-Phe [56]. In addition, Lund et al. proposed that Lac-Phe is an appetite suppressant and obesity-enhancing factor. Uric acid (UCA) is an endogenous component of the stratum corneum of human skin and is the major UV-absorbing component of the skin, usually accumulating in the trans form and isomerising from trans UCA (tUCA) to cis UCA (cUCA) when the skin is exposed to sunlight [57]. It is used as an intermediate in the histidine-to-glutamate metabolic pathway, mainly in mammalian livers or in bacteria, where it is normally synthesised by liver enzymes (e.g., hepatic enzymes) [58] and is converted to glutamate by the catalysis of a liver enzyme (urease). Myristic acid is a saturated fatty acid that is found in nature as a glyceride in vegetable fats and oils such as cardamom oil, palm oil, and coconut oil and is usually used in the formulation of various flavourings and additives, as well as in metal working [59]. In addition, by correlating myristic acid and type 2 diabetes, Wada et al. found that the reduced expression of diacylglycerol kinase (DGK) δ in skeletal muscle reduces the body’s glucose uptake, and it was found that myristic acid enhances the body’s insulin-dependent uptake of glucose and basal glucose uptake in the myotubes in a DGK δ2 expression-dependent manner [60]. In summary, different forms of exercise produce different metabolic changes in the organism. According to the results shown above, the differential metabolites found in the MICT group and the MICT+BFR group post exercise compared to those found pre- and post exercise in the MICT and MICT+BFR groups were consistent, including succinic acid, lactic acid, and N-lactoylphenylalanine (Lac-Phe). However, different metabolites were identified due to differences in metabolic pathways, e.g., citric acid, uric acid, and myristic acid [61,62,63]. In conclusion, there are similar differential metabolites but also different metabolomic profiles between MICT and MICT+BFR; therefore, the mechanisms underlying the differences produced by these two forms of exercise should be further explored in future studies.

Despite the fact that this study found different effects and mechanisms, there are still some limitations. First, this study was only conducted on a group of overweight/obese college students, making it less generalisable to other populations, and it is recommended that future research be expanded to a wider and more diverse population. Secondly, LC-MS/MS analysis of untargeted metabolomics was used in this study, which can comprehensively detect and analyse many types of metabolites but also leads to a lack of detection accuracy. Finally, this study only conducted a comparative metabolomics analysis of two types of exercise, namely MICT and MICT+BFR, and the comparative effects of metabolites between MICT and HIIT or between HIIT and MICT+BFR are not yet clear; subsequent studies can conduct metabolomics analyses and comparisons of many different forms of exercise to investigate the metabolic effects of different exercises on the body.

The subject sample size included in this study was small, and although we were limited by practical conditions, we have to acknowledge that a small sample size may have an effect on the experimental results. Therefore, we hope that future studies can use this as a basis to explore the results using a larger sample size or randomised controlled trial methods.

## 5. Conclusions

This study preliminarily suggests that MICT+BFR may be superior to MICT-only training in a lipid-lowering exercise intervention for obese college students, as significant increases in at least three lipolytic hormones, namely EPI, GH, and IL-6, were found following MICT+BFR training. Based on untargeted metabolomics analyses, it is suggested that the previously observed lipid-lowering effect of MICT+BFR was due to a more focused stimulation of the citric acid cycle and the alanine, aspartate, and glutamate metabolic pathways with this training modality.

## Figures and Tables

**Table 1:** Intra-group comparison of EPI levels in MICT, HIIT, and MICT+BFR groups.

Group	Pre-Exercise	Post-Exercise	Increase Range	Difference	t	*p*
M (*n* = 14)	73.04 ± 35.12	79.10 ± 42.09	8.3%	6.07 ± 8.76	2.59	0.022 *
H (*n* = 14)	75.15 ± 32.69	97.94 ± 44.38	30.4%	22.81 ± 16.12	5.29	0.000 **
B (*n* = 14)	72.81 ± 32.37	85.96 ± 40.67	18.1%	13.16 ± 16.56	2.97	0.011 *

Note: * indicates *p* < 0.05, i.e., the difference is significant compared to pre-exercise. ** indicates *p* < 0.01, i.e., the difference is highly significant compared to pre-exercise. M: MICT; H: HIIT; B: MICT+BFR; ±: standard deviation.

**Table 2:** Intra-group comparison of NA levels in MICT, HIIT, and MICT+BFR groups.

Group	Pre-Exercise	Post-Exercise	Increase Range	Difference	t	*p*
M (*n* = 14)	53.23 ± 78.31	55.81 ± 79.66	4.8%	2.56 ± 5.01	1.94	0.075
H (*n* = 14)	55.96 ± 85.77	60.09 ± 91.53	7.4%	4.14 ± 8.55	1.81	0.094
B (*n* = 14)	54.77 ± 76.49	60.06 ± 89.27	9.7%	5.30 ± 15.85	1.25	0.234

**Table 3:** Intra-group comparison of GH levels in MICT group, HIIT group, and MICT+BFR group.

Group	Pre-Exercise	Post-Exercise	Increase Range	Difference	t	*p*
M (*n* = 14)	206.16 ± 222.09	7027.94 ± 3988.48	3309%	6821.79 ± 3918.66	6.51	0.000 **
H (*n* = 14)	215.80 ± 235.62	8820.68 ± 5741.44	3988%	8604.86 ± 5737.05	5.61	0.000 **
B (*n* = 14)	219.13 ± 190.64	11,205.64 ± 5607.96	5014%	10,986.51 ± 5601.84	7.34	0.000 **

Note: ** indicates *p* < 0.01, i.e., the difference is highly significant compared with pre-exercise. ±: Standard deviation.

**Table 4:** Intra-group comparison of IL-6 levels between MICT group, HIIT group, and MICT+BFR group.

	Pre-Exercise	Post-Exercise	Increase Range	Difference	t	*p*
M (*n* = 14)	4.46 ± 1.73	5.11 ± 1.55	14.1%	0.64 ± 1.65	1.49	0.161
H (*n* = 14)	4.16 ± 2.02	5.63 ± 1.72	35.1%	1.44 ± 1.90	2.91	0.012 *
B (*n* = 14)	4.62 ± 2.10	7.04 ± 1.89	52.4%	2.42 ± 2.49	3.65	0.003 **

Note: * indicates *p* < 0.05, i.e., the difference is significant compared to pre-exercise. ** indicates *p* < 0.01, i.e., the difference is highly significant compared to pre-exercise. M: MICT; H: HIIT; B: MICT+BFR; ±: standard deviation.

## Data Availability

We encourage readers who need raw data to email the corresponding author and request the raw data.

## Supplementary Materials

The following supporting information can be downloaded at https://www.mdpi.com/article/10.3390/metabo14080433/s1: Supplementary Table S1. Comparison of EPI levels among MICT, HIIT, and MICT+BFR groups. Supplementary Table S2. Comparison of NA levels among MICT, HIIT, and MICT+BFR groups. Supplementary Table S3. Comparison of GH levels in MICT, HIIT, and MICT+BFR groups. Supplementary Table S4. Comparison of IL-6 levels among MICT, HIIT, and MICT+BFR groups. Supplementary Figure S1. PLS-DA overview graph for MICT group. Supplementary Figure S2. PLS-DA multivariate scores for MICT group. Supplementary Figure S3. VIP scores of MICT group. Supplementary Figure S4. Plasma metabolite hot spots before and after exercise in the MICT group. Supplementary Figure S5. Volcano plot of plasma metabolite differences before and after exercise in the MICT group. Supplementary Figure S6. Metabolic pathway bubble plots before and after exercise in the MICT group. Supplementary Figure S7. PLS-DA overview in MICT+BFR group. Supplementary Figure S8. PLS-DA multivariate statistical score in MICT+BFR group. Supplementary Figure S9. VIP scores in MICT+BFR group. Supplementary Figure S10. Plasma metabolite hot spots before and after exercise in the MICT+BFR group. Supplementary Figure S11. Volcano plot of plasma metabolite differences before and after exercise in MICT+BFR group. Supplementary Figure S12. Metabolic pathway bubble diagrams before and after exercise in the MICT+BFR group.
