# Supplementary material for: Deciphering Blood Flow Restriction Training to Aid Lipid Lowering in Obese College Students through Untargeted Metabolomics
Source: Metabolites. 2024 Aug 5;14(8):433. doi: 10.3390/metabo14080433 (PMC11356577; doi:10.3390/metabo14080433)
Supplement: Supplementary file 1 [file metabolites-14-00433-s001.zip › metabolites-3084016-supplementary.pdf]

Supplementary Tables

Supplementary Tables 1. Comparison of EPI levels among MICT, HIIT and MICT combined with BFR groups.

Supplementary Tables 2. Comparison of NA levels among MICT, HIIT and MICT combined with BFR groups.

Supplementary Tables 3. Comparison of GH levels in MICT, HIIT, and MICT combined with BFR groups.

Supplementary Tables 4. Comparison of IL-6 levels among MICT, HIIT and MICT combined with BFR groups.

Table S1. Comparison of EPI levels among MICT, HIIT and MICT combined with BFR groups.

Test metrics	分 Group		F	P
EPI（pg/ml）	MGroup(n=14)	HGroup(n=14)	20.920	0.001**
		BGroup(n=14)	4.463	0.055
	HGroup(n=14)	BGroup(n=14)	2.851	0.115

Table S2：Comparison of NA levels among MICT, HIIT and MICT combined with BFR groups

Test metrics	分 Group		F	P
NA（pg/ml）	MGroup(n=14)	HGroup(n=14)	0.431	0.523
		BGroup(n=14)	0.343	0.568
	HGroup(n=14)	BGroup(n=14)	0.094	0.763

Table S3：Comparison of GH levels in MICT, HIIT, and MICT combined with BFR groups

Test metrics	分 Group		F	P
GH（pg/ml）	MGroup(n=14)	HGroup(n=14)	1.893	0.192
		BGroup(n=14)	29.622	0.000**
	HGroup(n=14)	BGroup(n=14)	2.841	0.116

Table S4： Comparison of IL-6 levels among MICT, HIIT and MICT combined with BFR groups

Test metrics	分 Group		F	P
IL-6（pg/ml）	MGroup(n=14)	HGroup(n=14)	2.506	0.137
		BGroup(n=14)	3.812	0.073
	HGroup(n=14)	BGroup(n=14)	1.410	0.256

Supplementary figure 1. PLS-DA overview graph for MICT group
Supplementary figure 2. PLS-DA multivariate scores for MICT group
Supplementary figure 3. VIP scores of MICT group.
Supplementary figure 4. Plasma metabolite hotspots before and after exercise in the MICT group.
Supplementary figure 5. Volcano plot of plasma metabolite differences before and after exercise in the MICT group.
Supplementary figure 6. Metabolic pathway bubble plots before and after exercise in the MICT group.
Supplementary figure 7. PLS-DA overview in MICT+BFR group
Supplementary figure 8. PLS-DA multivariate statistical score in MICT+BFR group
Supplementary figure 9. VIP scores in MICT+BFR group
Supplementary figure 10. Plasma metabolite hotspots before and after exercise in the MICT+BFR group.
Supplementary figure 11.Volcano plot of plasma metabolite differences before and after exercise in MICT+BFR group
Supplementary figure 12. Metabolic pathway bubble diagrams before and after exercise in the MICT+BFR group.

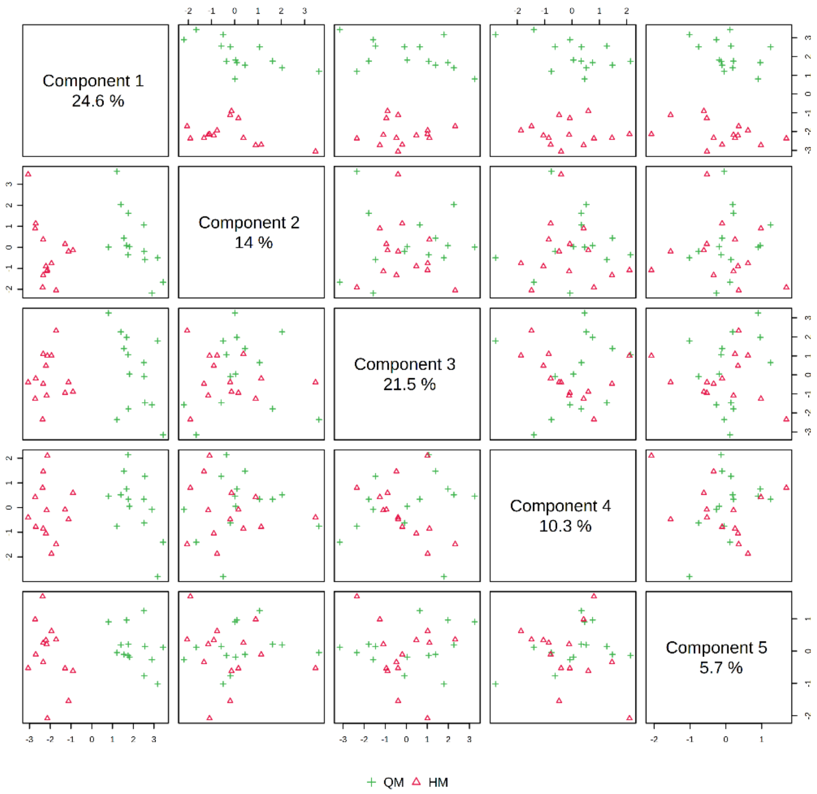

FigureS1. PLS-DA overview graph for MICT group

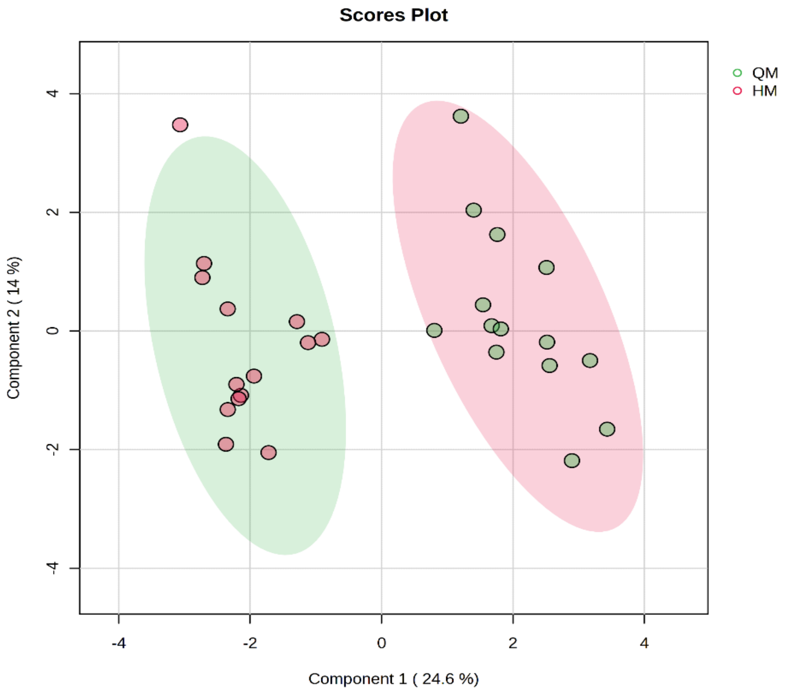

FigureS2. PLS-DA multivariate scores for MICT group

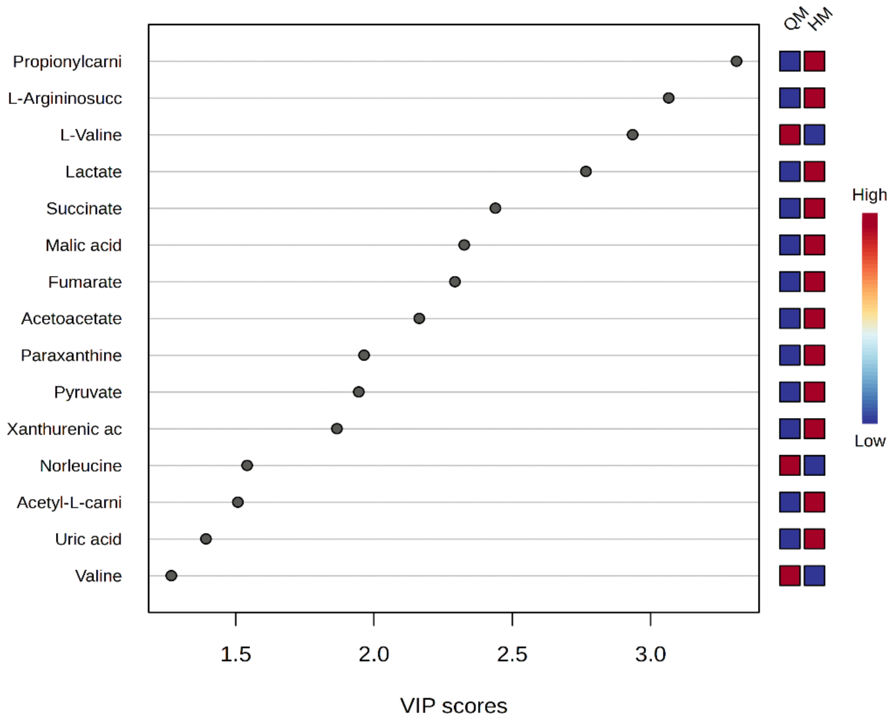

FigureS3. VIP scores of MICT group

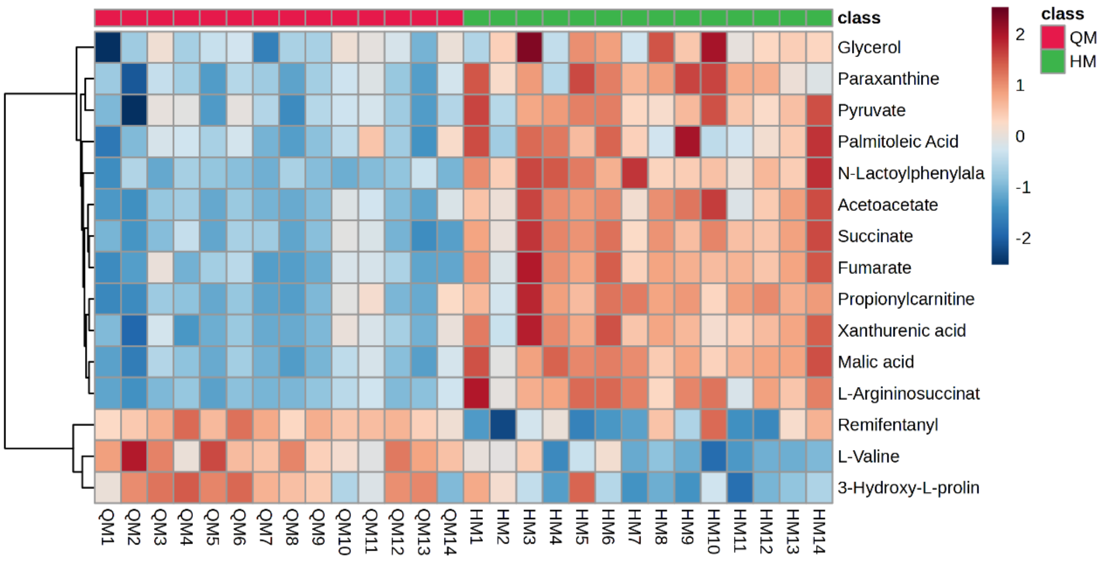

FigureS4. Plasma metabolite hotspots before and after exercise in the MICT group

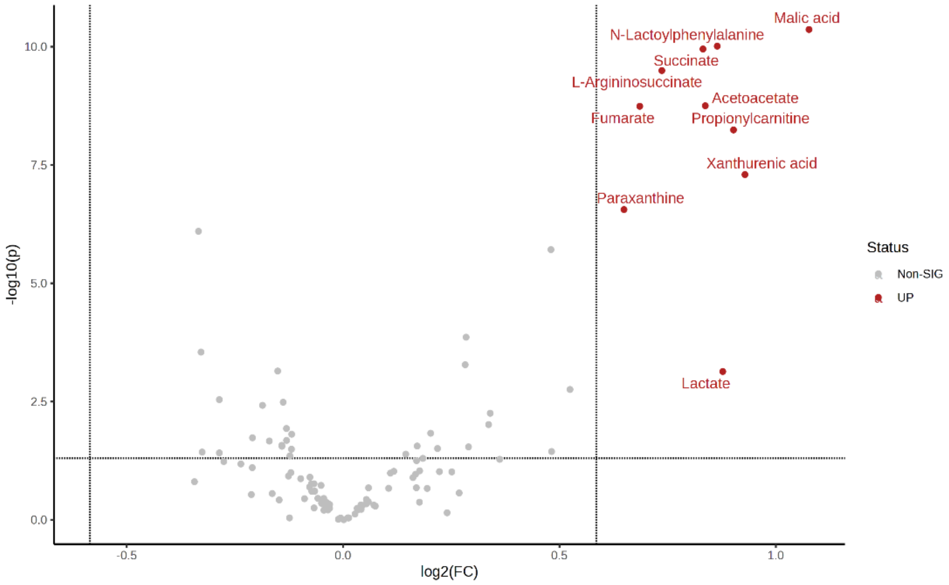

FigureS5. Volcano plot of plasma metabolite differences before and after exercise in the MICT group

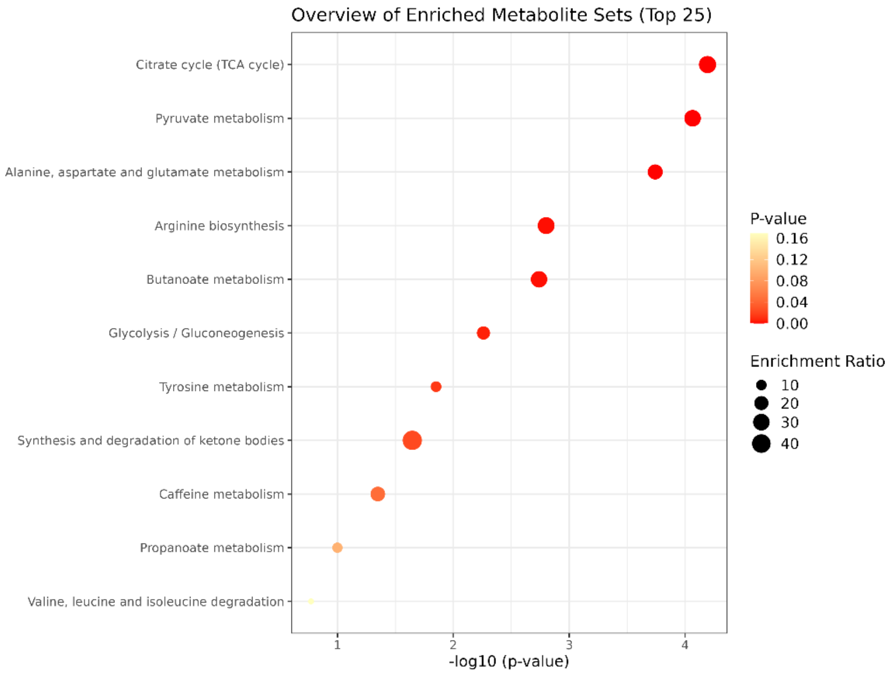

FigureS6. Metabolic pathway bubble plots before and after exercise in the MICT group.

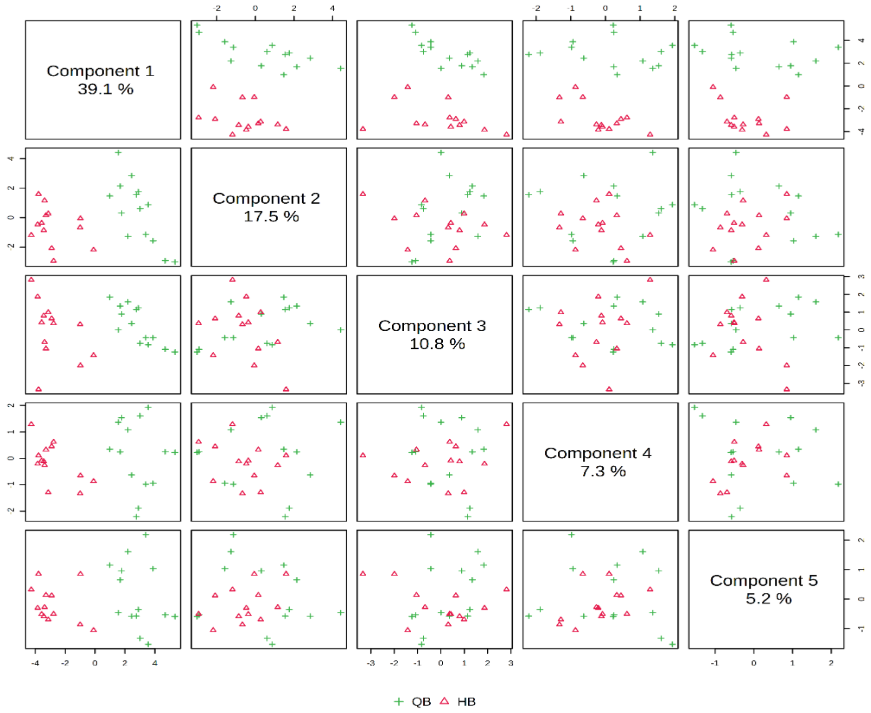

FigureS7. PLS-DA overview in MICT+BFR group

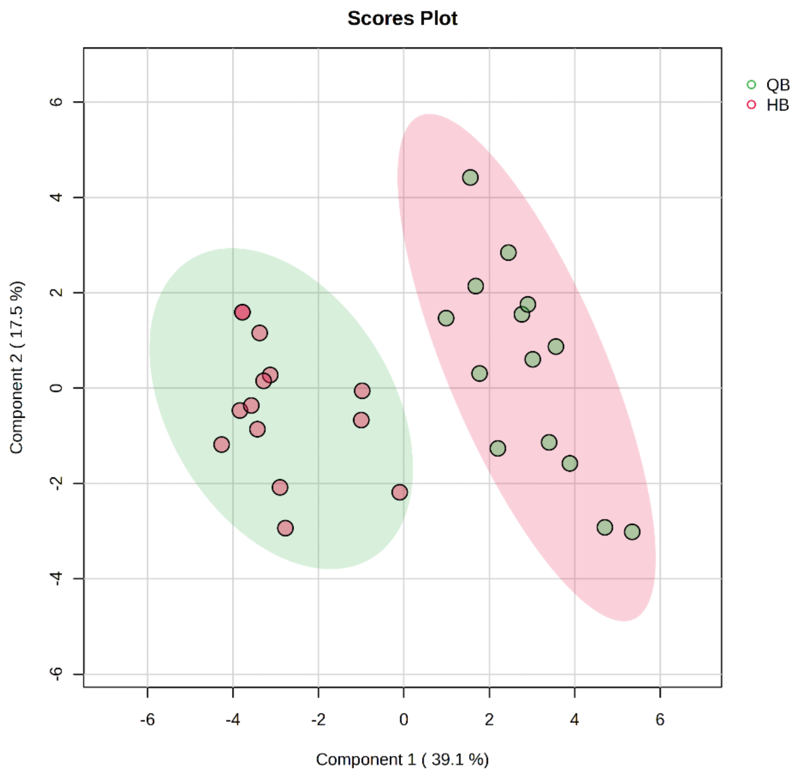

FigureS8. PLS-DA multivariate statistical score in MICT+BFR group

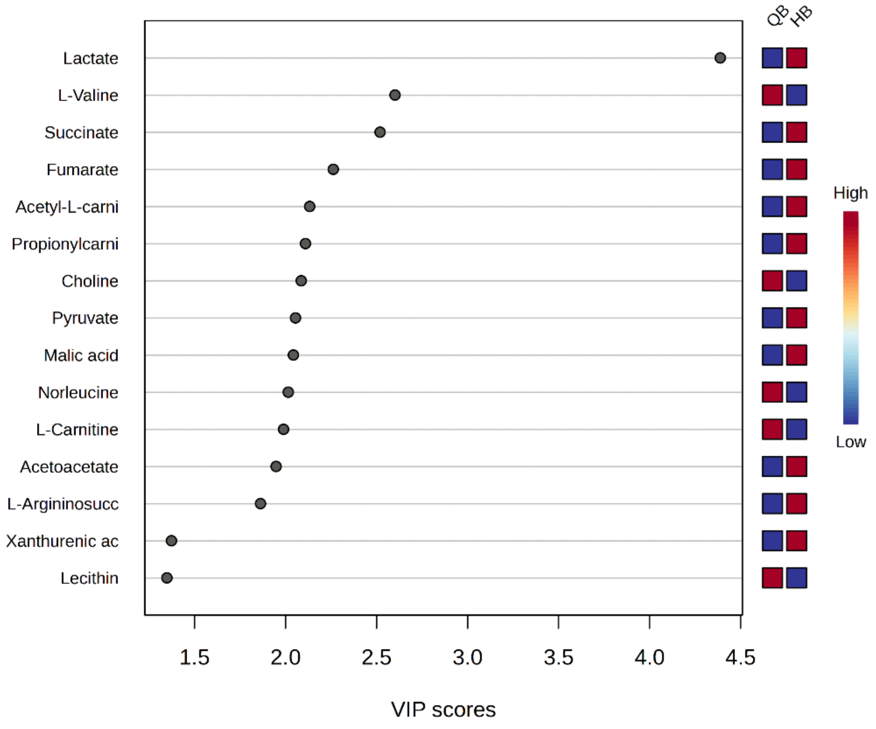

FigureS9. VIP scores in MICT+BFR group

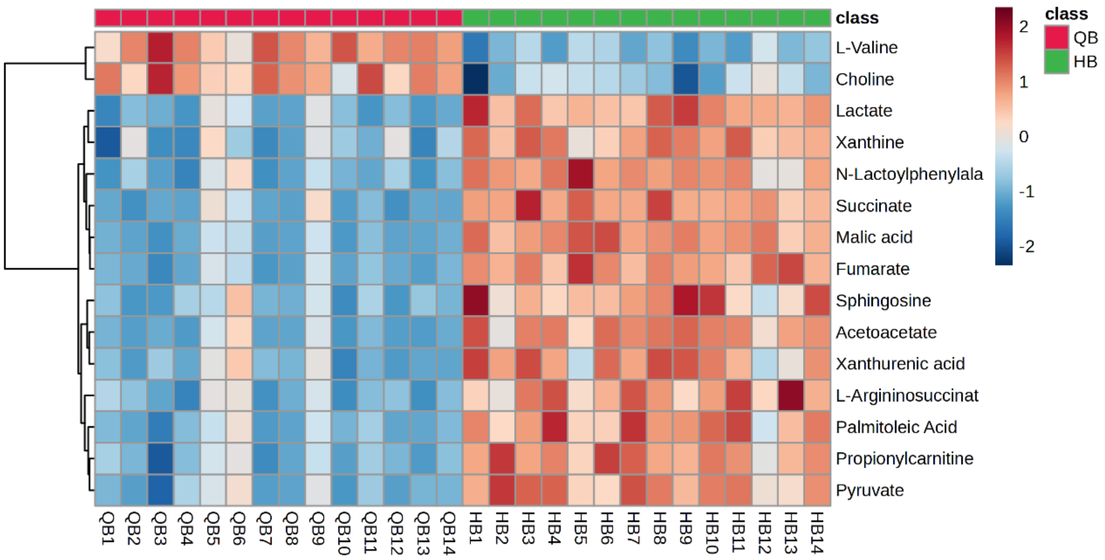

FigureS10. Plasma metabolite hotspots before and after exercise in the MICT+BFR group.

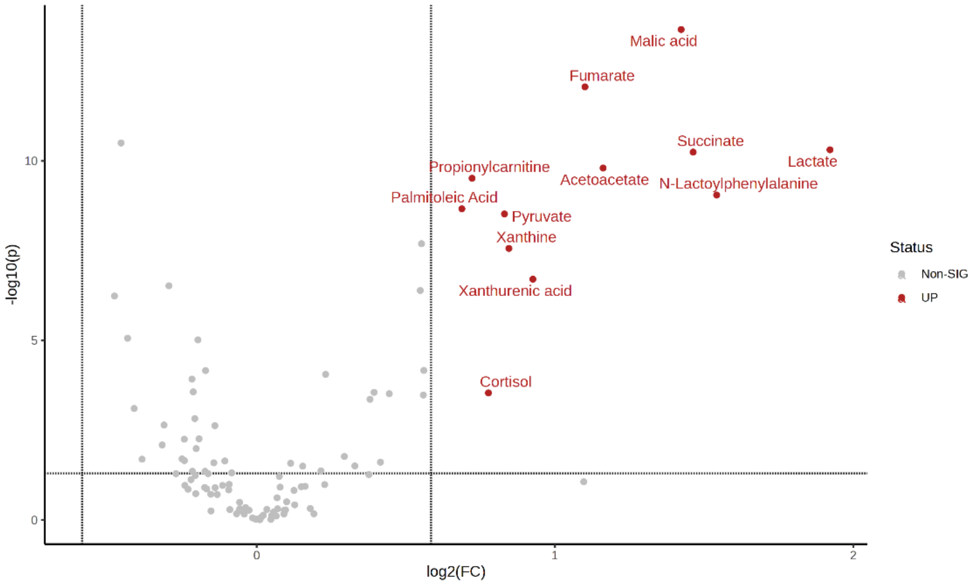

FigureS11. Volcano plot of plasma metabolite differences before and after exercise in MICT+BFR group

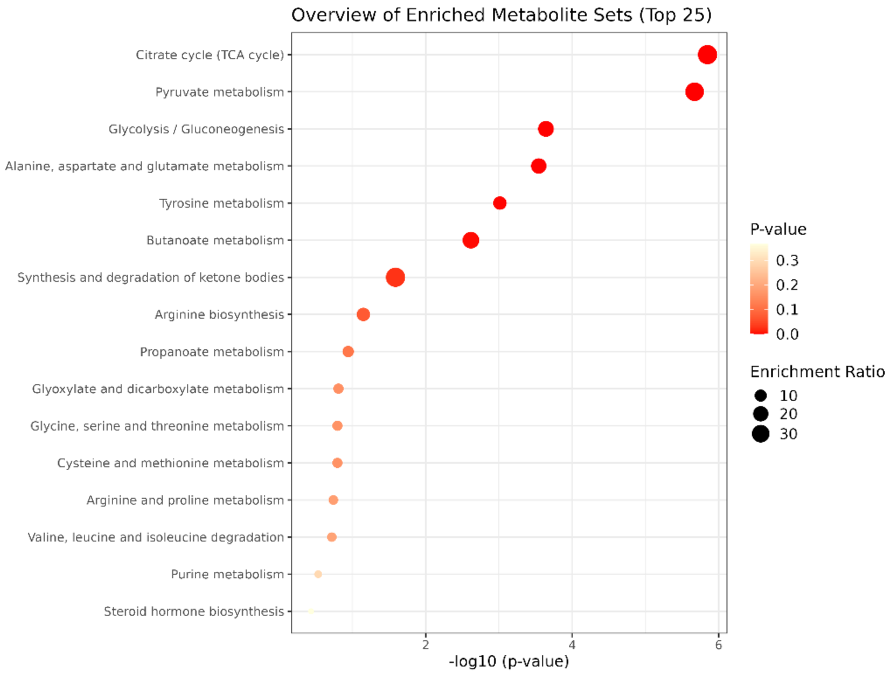

FigureS12. Metabolic pathway bubble diagrams before and after exercise in the MICT+BFR group.
